# Expression of p52, a non-canonical NF-kappaB transcription factor, is associated with poor ovarian cancer prognosis

**DOI:** 10.1186/s40364-020-00227-y

**Published:** 2020-09-15

**Authors:** Demetra H. Hufnagel, Andrew J. Wilson, Jamie Saxon, Timothy S. Blackwell, Jaclyn Watkins, Dineo Khabele, Marta A. Crispens, Fiona E. Yull, Alicia Beeghly-Fadiel

**Affiliations:** 1 Vanderbilt University School of Medicine, Nashville, TN 37240 USA; 2 Department of Obstetrics and Gynecology, Division of Gynecologic Oncology, Vanderbilt University Medical Center, Nashville, TN 37232 USA; 3 Vanderbilt-Ingram Cancer Center, Nashville, TN 37232 USA; 4 Division of Allergy, Pulmonary and Critical Care Medicine, Department of Medicine, Vanderbilt University Medical Center, Nashville, TN 37232 USA; 5 Department of Pathology, Microbiology, and Immunology, Vanderbilt University Medical Center, Nashville, TN 37232 USA; 6 Department of Obstetrics and Gynecology, Division of Gynecologic Oncology, Washington University School of Medicine, St. Louis, MO 63130 USA; 7 Department of Pharmacology, Vanderbilt University, Nashville, TN 37232 USA; 8 Division of Epidemiology, Department of Medicine, Vanderbilt University Medical Center, Nashville, TN 37203 USA

**Keywords:** NF-kappaB, Ovarian cancer, Survival, Prognosis

## Abstract

**Background:**

The canonical and non-canonical nuclear factor-kappaB (NF-κB) signaling pathways have key roles in cancer, but studies have previously evaluated only the association of canonical transcription factors and ovarian cancer survival. Although a number of in vitro and in vivo studies have demonstrated mechanisms by which non-canonical NF-κB signaling potentially contributes to ovarian cancer progression, a prognostic association has yet to be shown in the clinical context.

**Methods:**

We assayed p65 and p52 (major components of the canonical and non-canonical NF-κB pathways) by immunohistochemistry in epithelial ovarian tumor samples; nuclear and cytoplasmic staining were semi-quantified by H-scores and dichotomized at median values. Associations of p65 and p52 with progression-free survival (PFS) and overall survival (OS) were quantified by Hazard Ratios (HR) from proportional-hazards regression.

**Results:**

Among 196 cases, median p52 and p65 H-scores were higher in high-grade serous cancers. Multivariable regression models indicated that higher p52 was associated with higher hazards of disease progression (cytoplasmic HR: 1.54; nuclear HR: 1.67) and death (cytoplasmic HR: 1.53; nuclear HR: 1.49), while higher nuclear p65 was associated with only a higher hazard of disease progression (HR: 1.40) in unadjusted models. When cytoplasmic and nuclear staining were combined, p52 remained significantly associated with increased hazards of disease progression (HR: 1.91, *p* = 0.004) and death (HR: 1.70, *p* = 0.021), even after adjustment for p65 and in analyses among only high-grade serous tumors.

**Conclusions:**

This is the first study to demonstrate that p52, a major component of non-canonical NF-κB signaling, may be an independent prognostic factor for epithelial ovarian cancer, particularly high-grade serous ovarian cancer. Approaches to inhibit non-canonical NF-κB signaling should be explored as novel ovarian cancer therapies are needed.

## Background

Ovarian cancer is the 5th leading cause of cancer deaths among women in the United States and has the highest mortality among gynecologic cancers, with an overall 5-year survival rate lower than 50% [1]. While overall rates of death from cancer have dramatically declined over the past few decades, mortality from ovarian cancer has hovered around 10 women per 100,000 for the last 50 years [2, 3]. Long-term survival is poor as patients are most often diagnosed at advanced stage, largely due to non-specific symptoms and lack of early detection methods [4, 5]. Furthermore, while most patients have a dramatic initial response to first-line platinum and taxane-based chemotherapies, the majority will ultimately develop recurrent and treatment-resistant disease [1, 6]. This is particularly relevant to high-grade serous ovarian cancer, the most common and deadliest of the five main subtypes when classified by grade and histology: high-grade serous, low-grade serous, endometrioid, clear cell, and mucinous tumors [7–10]. Targeted treatments aimed at specific pro-tumorigenic gene products, such as vascular endothelial growth factor (VEGF) inhibitors and poly ADP ribose polymerase inhibitors (PARPi), are now being incorporated into the clinic as first-line maintenance therapies and treatment for recurrence [11–14]. The development of additional treatment modalities to improve ovarian cancer survival requires further understanding of the pathogenesis of this disease.

Activation of nuclear factor-kappaB (NF-κB) signaling has been identified as an important molecular link between inflammation and cancer, and modulates tumor growth, chemotherapy resistance, and immune escape in a variety of malignancies, including ovarian cancer [15–17]. NF-κB signaling is mediated by dimerization of a family of five transcription factors that share N-terminal DNA binding Rel homology domains: p50 (and its precursor p105), p52 (and its precursor p100), p65/RelA, RelB, and c-Rel. RelA, RelB, and c-Rel additionally contain C-terminal nuclear localization signals that facilitate nuclear translocation [18]. In homeostatic conditions, homo- and heterodimers of NF-κB members are bound to inhibitor of kappaB proteins (IκB) and sequestered in the cytoplasm. Following upstream NF-κB pathway signaling, inhibitor of kappaB kinase (IKK) proteins phosphorylate IκB, causing its proteasomal degradation and release of the dimer from inhibition, thereby allowing for nuclear translocation and transcriptional activation of anti-apoptotic and pro-proliferative gene targets [18].

NF-κB signaling can be divided into two broad pathways, termed canonical and non-canonical. Activation of canonical NF-κB signaling occurs when pro-inflammatory ligands bind to cell surface receptors such as tumor necrosis factor receptors or Toll-like receptors, most often resulting in nuclear localization of p65/p50 dimers to initiate transcription of acute phase reactants, inflammatory cytokines, and regulators of apoptosis [18]. Non-canonical NF-κB signaling typically occurs following binding of a ligand to the CD40 receptor, lymphotoxin beta receptor, or B-cell activating factor receptor. Activation of this cell surface receptor results in phosphorylation of NF-κB-inducing kinase (NIK), which then activates IKK to induce processing of cytoplasmic p100 to active p52 with resultant nuclear localization of p52/RelB dimers [19].

A few studies have evaluated the expression of p65 and other canonical NF-κB pathway members in ovarian cancer, but findings for associations with patient outcomes have been inconsistent [20–25]. Associations between high p65 expression and poor ovarian cancer survival have been reported [20, 24]; however, the largest study of human tissue found better overall survival for high nuclear p65 expression among 324 high-grade serous cases [21]. Non-canonical NF-κB signaling remains relatively understudied compared to canonical signaling and comparatively little is known about the non-canonical NF-κB pathway and cancer survival. Prior studies have demonstrated increased activation of p52 in breast, lung, prostate, pancreatic, and ovarian cancers [26–32], and increased expression has been linked to worse prognosis in lung cancer [33]. While pre-clinical in vitro and in vivo studies have demonstrated a variety of mechanisms by which non-canonical NF-κB signaling may contribute to ovarian cancer carcinogenesis and progression, no studies to date have reported associations between non-canonical NF-κB transcription factors and ovarian cancer clinical prognosis [31, 32, 34, 35]. Thus, we undertook this study to evaluate if variation in expression of NF-κB transcription factors, specifically tumor expression of p65 (a major contributor to canonical signaling) and p52 (a major contributor to non-canonical signaling), was associated with survival outcomes among a retrospective cohort of ovarian cancer cases.

## Materials and methods

### Institutional approval

Approval was previously obtained from the Vanderbilt University Medical Center (VUMC) Institutional Review Board for construction of a tissue microarray (TMA) and collection of clinical data for Gynecologic Oncology patients at VUMC from 1994 to 2004; as all patient data was de-identified and discarded tissue from routine clinical care was evaluated, we obtained a waiver of consent from the VUMC Institutional Review Board committee. Data collection and processing were performed in accordance with standard guidelines, including US Federal Policy for the Protection of Human Subjects and the Declaration of Helsinki.

### Ovarian cancer tissue microarray

We evaluated a validated TMA constructed from primary ovarian tumor samples from the VUMC Tissue Repository for Ovarian Cancer (TROC) [36, 37]. Briefly, tissue samples were collected from patients seeking care at VUMC at the time of primary staging and/or cytoreductive surgical procedures by the Division of Gynecologic Oncology between 1994 and 2004. Inclusion criteria included age > 18 years and a primary epithelial tumor of the ovary. Patient samples were excluded if they were non-epithelial ovarian tumors or were non-primary (i.e. metastatic or recurrent) tumor of the ovary (Additional File 1). Hematoxylin and eosin stained sections from identified cases were reviewed and a representative formalin-fixed paraffin-embedded tissue block containing > 80% tumor cells was identified for each case. For each specimen, four 1 mm cores from the representative tumor block were included in the tissue microarray. Tumor cores with < 100 tumor cell nuclei were excluded from analysis. Because ovarian cancer classification has been updated over time, our samples underwent additional pathology review to re-classify cases according to the contemporary 2014 WHO guidelines [8, 38]. Among the 196 epithelial samples included the current analysis, there were 177 invasive tumors of serous (*N* = 131), endometrioid (*N* = 24), mucinous (*N* = 8), clear cell (*N* = 11), and other tumor histotypes (*N* = 3: a carcinosarcoma, a primary squamous cell carcinoma, and a small cell carcinoma); a total of 19 samples were from non-invasive borderline tumors of either serous (*N* = 12) or mucinous (*N* = 7) histology.

### Clinical database

Demographic and clinical information, including stage, grade, treatment history, and patient outcomes were obtained by manual data abstraction from the VUMC electronic medical record (EMR) and collected using a REDCap database built for the VUMC TROC [39]. Tumor stage was classified based on the International Federation of Gynecology and Obstetrics (FIGO) system, with early stage including stages I and II and late stage including III and IV [40]. Low-grade tumors included low-grade serous tumors and grade 1 and 2 endometrioid and mucinous tumors; high-grade tumors included high-grade serous carcinomas, grade 3 endometrioid and mucinous tumors, clear cell carcinomas, and carcinosarcomas. Date of diagnosis was defined as the earliest date of pathologic confirmation, either by cytologic evidence of adenocarcinoma from ascites or from pathologic evaluation of tissue specimen. Date of disease progression or recurrence after staging and/or debulking surgery in the first-line treatment setting was defined by earliest evidence of increasing measurable disease, as determined by RECIST imaging criteria, development of new or enlarged lesions on physical exam, confirmed ovarian cancer on biopsy of new lesions, or disease progression explicitly stated within the EMR [41]. Date of death was ascertained from VUMC EMR, Tumor Registry, OnCore Clinical Trial Research Databases, and the National Technical Information Services Death Master File of deaths reported to the Social Security Administration. Outcomes were captured through 11/29/2018. Patients without visible pelvic extension of disease at the time of primary surgery were categorized as “not applicable” for debulking. Among patients with advanced pelvic disease, 5 had unresectable disease and were not debulked, and 24 had unknown residual disease. For those who underwent debulking with outcomes known, debulking was characterized in accordance with current guidelines based on manual review of operative reports as optimal (not otherwise specified, no residual disease, residual disease ≤ 1 cm maximum), or sub-optimal (> 1 cm) [42]. Six samples were obtained during interval debulking after neoadjuvant chemotherapy, while 190 samples were obtained during primary tumor reductive surgery prior to any adjuvant chemotherapy and were classified as “chemotherapy naïve.” All chemotherapy data was collected from first-line treatment. Among patients who had dates of first-line platinum chemotherapy available, platinum sensitivity was defined as a progression-free interval of greater than 6 months after completion of first-line chemotherapy, while platinum resistance was defined by a progression-free interval of less than 6 months following completion of first-line chemotherapy. Accordingly, patients with resistant or refractory disease (demonstrated progression at the time of first-line therapy) were grouped together for this study. Patients for whom the date of completion of first-line platinum chemotherapy was unknown were categorized as unknown for response to platinum treatment.

### Immunohistochemistry (IHC)

We conducted immunostaining for p52 with anti-p52 (C-5, Santa Cruz; sc-7386) on TMA slides utilizing a previously published protocol [33]. Staining of p65 was performed under automated conditions by the Vanderbilt Translational Pathology Shared Resource with mouse monoclonal anti-p65 (F-6, Santa Cruz; sc-8008). Human tonsil, lung parenchyma, and lung adenocarcinoma, were used as positive controls. True negative controls were not feasible as NF-κB signaling is ubiquitous in human tissue. However, we selected commercially available antibodies that have been well-studied and validated in prior IHC studies of NF-κB transcription factors in a variety of human tissues [33, 43]. Whole-slide imaging and semi-quantitative measurement of the percentage of epithelial tumor cells with positive cytoplasmic and nuclear expression was performed using the automated Ariol SL-50 Platform in the Digital Histology Shared Resource of the VUMC. Cytoplasmic and nuclear H-scores were calculated for each sample by multiplying the percentage of cells staining positive (0–100) by staining intensity (weak: 1, moderate: 2, or strong: 3), to yield an H-score ranging from 0 to 300. Low and high expression of all markers was dichotomized a priori by median values so that our exposure of interest, expression of p52 or p65, was not defined by our outcome of interest, disease prognosis.

### Statistical analysis

Normality of continuous variables, including H-scores for IHC quantification, was visually assessed using histograms; based on this and our sample sizes, we generally used non-parametric statistical approaches. Monotonic correlations between continuous variables were calculated using Spearman’s rank correlation coefficient, and the Kruskal-Wallis test was used to compare two or more groups of non-normal data, such as differences in NF-κB expression in relation to clinical covariates. The chi-squared test was used to assess relationships between categorical variables. In line with prior studies of NF-κB pathway effectors in ovarian cancer tissue, we also performed analyses where nuclear and cytoplasmic staining were combined [23]; if either was higher than the median, then expression was considered to be high. Overall survival (OS) was defined as the interval between date of diagnosis to either date of death or censored at the date of last contact. Progression-free survival (PFS) was defined as the interval from date of diagnosis to date of first disease progression after initial staging and/or cytoreductive surgery in the first-line treatment setting, death, or censored at the date of last contact. To minimize potential bias from loss to follow-up, all events after were censored and time was truncated at 15-years post-diagnosis. Survival functions were visualized using Kaplan-Meier plots and differences were assessed by Log-Rank tests. Associations with survival outcomes were quantified using hazard ratios (HR) and 95% confidence intervals (CI) calculated from Cox proportional-hazards regression with calendar time as the time-scale. Adjustment for clinical covariates included known prognostic factors as well as significant associations from our bivariate analysis: age at diagnosis, stage, histologic subtype, grade, and platinum and/or taxane chemotherapy treatment. Maximal adjustment additionally included race, residual disease after debulking, and response to platinum therapy. Combined staining indices enabled us to employ mutually adjusted regression models to disentangle the effects of canonical and non-canonical NF-κB expression. To evaluate the robustness of our findings among all cases (*N* = 196), we conducted sensitivity analyses among chemotherapy naïve cases (*N* = 190), invasive cases (*N* = 177), serous cases (*N* = 143), late stage cases (*N* = 137), invasive serous cases (*N* = 131), late stage serous cases (*N* = 119) and high-grade serous cases (*N* = 118). Analyses were conducted in R version 3.4.4 using the *ggplot2* and *survminer* packages, and with the SAS System for Windows (SAS version 9.4) [44–47]. A *p*-value ≤ 0.05 was interpreted as statistically significant, with a Bonferroni corrected significance threshold used when considering multiple comparisons.

## Results

### Baseline characteristics

Among 196 cases from the Vanderbilt TROC TMA, clinical characteristics followed expected distributions (Table 1). Cases had a median age of diagnosis of 58.3 years, median progression-free survival of 1.6 years, and median overall survival of 3.6 years. The majority of cases evaluated were White (91.8%) with advanced stage (70.3%), high-grade (73.5%), serous histology (72.9%), and most were treated with chemotherapy (77.6%). Among 152 patients who received first-line chemotherapy, 150 patients (98.6%) had documentation of receiving platinum agents, and 147 patients (96.7%) had documentation of receiving both platinum and taxane agents. Two patients were noted to have completed first-line chemotherapy, but did not have specific therapeutic agent information available and were assumed to have had at least platinum therapy. A total of 44 patients either declined chemotherapy or did not have any information regarding possible first-line chemotherapy available, the majority of which (66%) had stage I disease.

**Table 1:** Patient and clinical characteristics among 196 ovarian tumors from the VUMC TROC

Characteristic	N or median	% * or std dev
**Age at diagnosis**, years	58.3	(13.9)
**Progression-free survival**, years	1.6	(3.8)
**Overall survival**, years	3.6	(6.1)
**Race**		
White	180	(91.8)
Black	12	(6.1)
Other	3	(1.5)
Unknown	1	(0.51)
**Stage**		
I	50	(25.6)
II	8	(4.1)
III	116	(59.5)
IV	21	(10.8)
Unknown	1	(0.5)
**Histologic subtype**		
Serous	143	(72.9)
Endometrioid	24	(12.2)
Mucinous	15	(7.7)
Clear cell	11	(5.6)
Other ^a^	3	(1.5)
**Grade - among all types** ^**b**^		
Borderline	19	(9.7)
Low-grade	33	(16.8)
High-grade	144	(73.5)
**Grade - among invasive serous**		
Low-grade	13	(9.9)
High-grade	118	(90.1)
**Debulking status**		
Debulked - optimal debulking	60	(30.6)
Debulked - suboptimal debulking	53	(27.0)
Not debulked or residual unknown	29	(14.8)
Not applicable	54	(27.6)
**Chemotherapy treatment**		
Platinum and/or taxane agent(s)	152	(77.6)
None or unknown	44	(22.4)
**Response to platinum treatment** ^**c**^		
Platinum sensitive	105	(70.0)
Platinum resistant or refractory	30	(20.0)
Unknown	15	(10.0)

* Percentages may not sum to 100 due to rounding error

^a^ Includes one case each: carcinosarcoma, primary squamous cell carcinoma, and a small cell carcinoma

^b^ High-grade includes high-grade serous, grade 3 endometrioid and mucinous, and all clear cell and carcinosarcomas. Low-grade includes low-grade serous, and grade 1–2 endometrioid and mucinous cancers

^c^ Among patients who had documentation of receiving platinum chemotherapy (*N* = 150)

### Expression in epithelial ovarian tumors

**Fig. 1:**
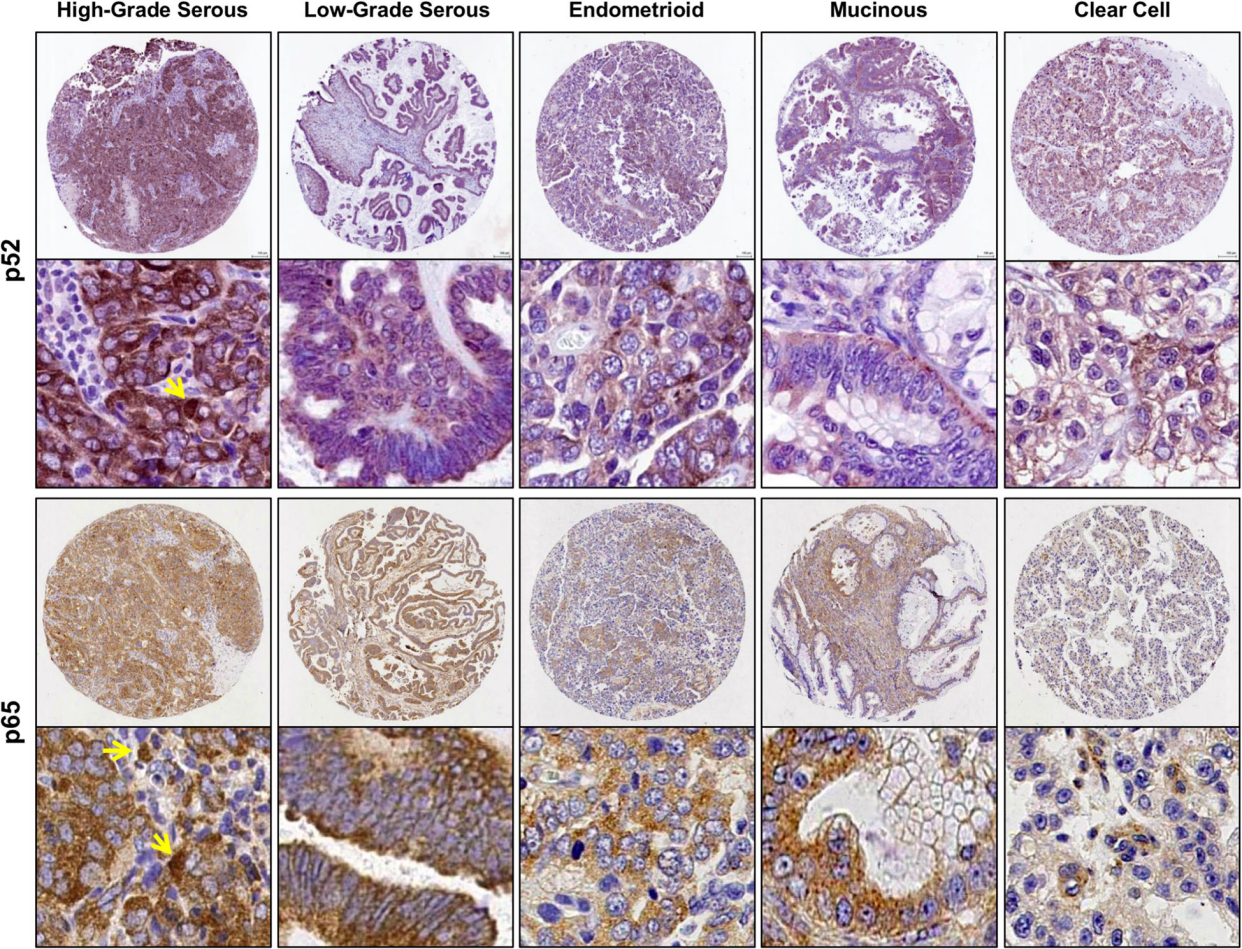
Representative IHC staining for p52 and p65 expression, the VUMC TROC. Representative immunohistochemical (IHC) staining for p52 and p65 in a variety of ovarian tumor histologic subtypes. Staining was predominantly cytoplasmic, with a smaller proportion of tumor cells showing nuclear staining (yellow arrows)

Expression of p52 and p65 was detected by immunohistochemistry (Fig. 1). Expression was predominantly cytoplasmic, with a relatively smaller proportion of tumor cells showing nuclear staining for both p52 (median H-scores: 135.9 vs. 1.0) and p65 (median H-scores: 154.3 vs. 2.7). To assess relationships between cytoplasmic and nuclear expression, we evaluated Spearman’s correlation coefficients. There was a strong positive monotonic correlation between cytoplasmic and nuclear staining for both p52 (rho = 0.69, *p* < 0.001) and p65 (rho = 0.78, *p* < 0.001). Correlations between canonical and non-canonical pathway members were much lower (cytoplasmic p65 and cytoplasmic p52, rho = 0.23; nuclear p65 and nuclear p52, rho = 0.25; both *p* < 0.001).

### Associations with clinical characteristics

Regardless of whether cytoplasmic or nuclear staining was assessed, median p52 H-scores were significantly higher among high-grade, advanced stage, and serous tumors, and among patients who were treated with chemotherapy (Table 2, *p* < 0.05). Similarly, regardless of whether cytoplasmic or nuclear staining was assessed, median p65 H-scores were significantly higher among high-grade, serous, and high-grade serous tumors (*p* < 0.05). Following Bonferroni correction for multiple comparisons, expression of p52 and p65 remained significantly higher among high-grade and serous cases (*P*-value ≤ 0.003571). When combined indices were assessed, the same associations with p52 remained significant as in compartmentalized analyses; combined high p52 expression was significantly associated with high grade, advanced stage, serous tumors, and receipt of platinum and/or taxane chemotherapy (all *p* ≤ 0.002, data not shown). As in compartmentalized analyses, combined high p65 expression was significantly associated with serous tumors (*p* < 0.001); associations with high-grade and high-grade serous tumors approached significance (*p* = 0.057, *p* = 0.076, respectively, data not shown). In all analyses, expression of p52 and p65 was not significantly associated with race, residual disease following surgical debulking, or platinum sensitivity.

**Table 2:** NF-κB staining by patient and tumor characteristics, the VUMC TROC

Characteristic	N	% *	p52 (Non-canonical NF-κB transcription factor)				p65 (Canonical NF-κB transcription factor)			
			Cytoplasmic		Nuclear		Cytoplasmic		Nuclear	
			Median	***P***-value **	Median	***P***-value **	Median	***P***-value **	Median	***P***-value **
**Race**										
White	180	(91.8)	135.9	0.698	1.0	0.815	156.1	0.956	2.7	0.831
Black, other, & unknown	16	(8.2)	122.8		1.0		144.0		2.4	
**Stage of disease** ^a^										
Early (I/II)	58	(29.6)	81.0	***< 0.001***	0.0	***< 0.001***	135.0	0.065	1.8	0.133
Late (III/IV)	137	(69.9)	164.8		1.8		158.6		3.0	
**Histologic subtype**										
Serous	143	(72.9)	152.0	***< 0.001***	2.2	***< 0.001***	165.4	***< 0.001***	3.9	***< 0.001***
Endometrioid & clear cell	35	(17.9)	97.7		0.0		118.8		0.4	
Mucinous & others	18	(9.2)	12.1		0.0		122.8		1.4	
**Grade - all cases**										
Borderline	19	(9.7)	38.3	***< 0.001***	0.0	**0.005**	123.4	**0.017**	1.6	***0.002***
Low-grade	33	(16.8)	103.6		0.4		127.4		0.0	
High-grade	144	(73.5)	157.8		1.6		160.0		3.9	
**Grade - invasive serous**										
Low-grade serous	13	(7.5)	117.2	0.057	1.0	0.237	146.0	**0.027**	1.4	**0.034**
High-grade serous	118	(67.8)	165.8		3.2		168.1		4.2	
**Residual disease** ^b^										
Optimal	60	(53.1)	121.4	0.102	1.6	0.723	157.3	0.970	2.6	0.191
Suboptimal	53	(46.9)	168.7		1.7		158.6		3.8	
**Chemotherapy**										
Platinum and/or taxane	152	(77.6)	152.0	***0.002***	1.6	**0.010**	156.5	0.090	2.8	0.145
None or unknown	44	(22.4)	91.4		0.0		144.7		1.8	
**Response to platinum** ^c^										
Platinum sensitive	105	(77.7)	152.0	0.538	1.6	0.558	161.4	0.771	2.8	0.808
Resistant or refractory	30	(22.2)	142.8		1.0		146.5		3.8	

* Column percentages may not sum to 100% due to rounding error

** Bold values denote significant differences by the Kruskal-Wallis test; those in italics denote that significance surpasses a Bonferroni corrected threshold for multiple comparisons (*P*-value ≤ 0.003571)

^a^ Excluding one case with unstaged disease

^b^ Among cases who had debulking surgery and outcomes ascertained

^c^ Among patients who had dates of first-line platinum chemotherapy available

### Analysis with survival outcomes

**Fig. 2:**
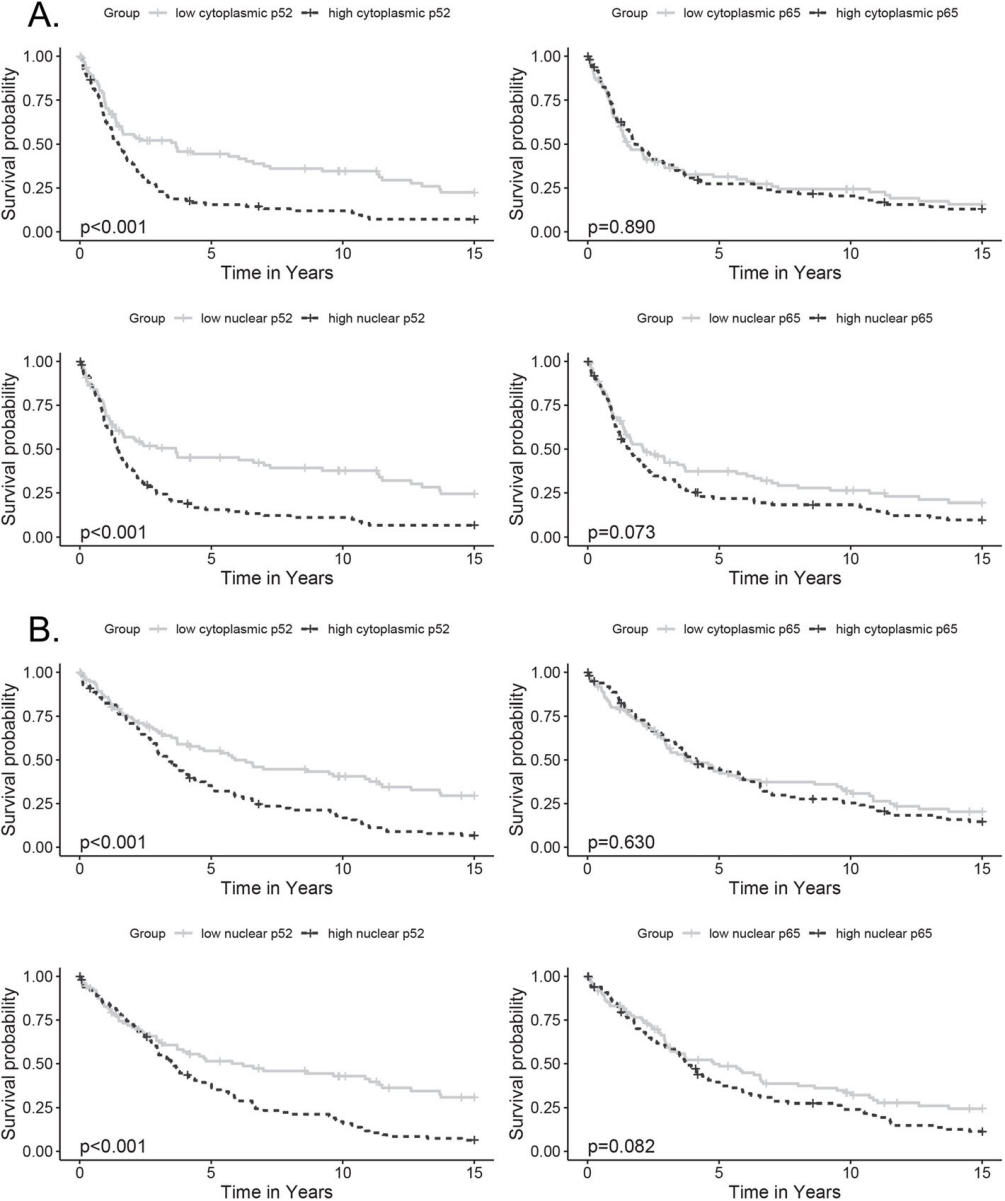
Kaplan-Meier survival functions for p52 and p65 expression, the VUMC TROC. Survival outcomes by p52 or p65 H-scores (dichotomized); **a**: progression-free survival (PFS); **b**: overall survival (OS); rows: cytoplasmic expression, nuclear expression; columns: p52 expression, p65 expression. Dotted line = median or higher expression; Solid line = lower than median expression. X-axis: survival time in years; Y-axis: progression-free or overall survival probability. *P*-values from Log-Rank tests

Kaplan-Meier analysis indicated that higher than median nuclear p52 expression was associated with significantly worse PFS and OS (Log-Rank *p* < 0.001) and that higher than median cytoplasmic p52 expression was similarly associated with significantly worse PFS and OS (Log-Rank *p* < 0.001) (Fig. 2a-b). In contrast, neither cytoplasmic nor nuclear expression of p65 were significantly associated with worse PFS or OS by Kaplan-Meier analysis.

In unadjusted proportional-hazards regression models, cases with high cytoplasmic p52 expression had approximately two-fold higher risks of disease progression (HR 2.15, 95% CI 1.55–2.98, *p* < 0.001) and death (HR 1.98, 95% CI 1.42–2.75, *p* < 0.001) (Table 3). Cases with high nuclear p52 expression similarly had approximately two-fold higher risk of disease progression (HR 2.05, 95% CI 1.46–2.86, *p* < 0.001) and death (HR 1.82, 95% CI 1.30–2.55, *p* < 0.001). Higher nuclear p65 was also associated with a 40% higher risk of disease progression (HR 1.40, 95% CI 1.02–1.92, *p* = 0.037) when covariates were not considered. In multivariable models adjusted for age at diagnosis, stage, histologic subtype, grade, and platinum and/or taxane chemotherapy treatment, all p52 associations remained significant; cases with higher than median cytoplasmic or nuclear p52 expression had approximately 60% higher risks of disease progression (cytoplasmic: HR 1.54, 95% CI 1.09–2.18, *p* = 0.015; nuclear: HR 1.67, 95% CI 1.15–2.42, *p* = 0.006) and approximately 50% higher risks of death (cytoplasmic: HR 1.53, 95% CI 1.07–2.18, *p* = 0.019; nuclear: HR 1.49, 95% CI 1.02–2.17, *p* = 0.041). In contrast, the association between higher nuclear p65 and worse PFS was attenuated after multivariable adjustment (nuclear: HR 1.20, 95% CI 0.85–1.69, *p* = 0.300). In models with maximal adjustment including age at diagnosis, stage, histologic subtype, grade, chemotherapy treatment, race, residual disease after debulking, and response to platinum therapy, our results were not substantially changed.

**Table 3:** NF-κB staining and ovarian tumor survival outcomes, the VUMC TROC

	Progression-free survival (PFS)				Overall survival (OS)			
	Unadjusted		Adjusted ^**a**^		Unadjusted		Adjusted ^**a**^	
**Compartmentalized staining**	**HR (95% CI)**	***P*-value**	**HR (95% CI)**	***P*-value**	**HR (95% CI)**	***P*-value**	**HR (95% CI)**	***P*-value**
**p52: Non-canonical NF-κB transcription factor**								
Cytoplasmic staining	**2.15 (1.55–2.98)**	**< 0.001**	**1.54 (1.09–2.18)**	**0.015**	**1.98 (1.42–2.75)**	**< 0.001**	**1.53 (1.07–2.18)**	**0.019**
Nuclear staining	**2.05 (1.46–2.86)**	**< 0.001**	**1.67 (1.15–2.42)**	**0.006**	**1.82 (1.30–2.55)**	**< 0.001**	**1.49 (1.02–2.17)**	**0.041**
**p65: Canonical NF-κB transcription factor**								
Cytoplasmic staining	1.02 (0.75–1.40)	0.885	1.05 (0.75–1.47)	0.776	1.08 (0.79–1.48)	0.636	1.13 (0.81–1.57)	0.472
Nuclear staining	**1.40 (1.02–1.92)**	**0.037**	1.20 (0.85–1.69)	0.300	1.37 (1.00–1.89)	0.052	1.31 (0.93–1.84)	0.124
**Total staining**								
**Independent models**								
p52: Cytoplasmic or Nuclear	**1.78 (1.28–2.47)**	**< 0.001**	**1.53 (1.08–2.19)**	**0.018**	**1.73 (1.24–2.41)**	**0.001**	**1.61 (1.12–2.33)**	**0.011**
p65: Cytoplasmic or Nuclear	1.38 (0.99–1.92)	0.054	1.27 (0.89–1.81)	0.186	1.38 (0.99–1.92)	0.059	1.31 (0.92–1.88)	0.138
**Mutually adjusted models** ^**b**^								
p52: Cytoplasmic or Nuclear	**1.70 (1.21–2.38)**	**0.002**	**1.49 (1.04–2.14)**	**0.030**	**1.64 (1.16–2.32)**	**0.005**	**1.57 (1.08–2.27)**	**0.018**
p65: Cytoplasmic or Nuclear	1.24 (0.88–1.73)	0.217	1.18 (0.82–1.71)	0.348	1.22 (0.86–1.72)	0.260	1.23 (0.85–1.77)	0.265
**Among high-grade serous cases** ^**c**^								
**Independent models**								
p52: Cytoplasmic or Nuclear	**1.79 (1.17–2.75)**	**0.007**	**1.68 (1.08–2.62)**	**0.020**	**1.65 (1.07–2.56)**	**0.024**	**1.61 (1.03–2.53)**	**0.038**
p65: Cytoplasmic or Nuclear	0.90 (0.58–1.36)	0.606	1.14 (0.74–1.76)	0.551	1.02 (0.67–1.57)	0.921	1.16 (0.75–1.81)	0.499
**Mutually adjusted models** ^**b**^								
p52: Cytoplasmic or Nuclear	**1.91 (1.23–2.96)**	**0.004**	**1.68 (1.07–2.62)**	**0.024**	**1.70 (1.09–2.66)**	**0.021**	**1.59 (1.01–2.52)**	**0.045**
p65: Cytoplasmic or Nuclear	0.76 (0.49–1.18)	0.223	1.03 (0.66–1.61)	0.899	0.89 (0.57–1.39)	0.612	1.09 (0.69–1.69)	0.719

^a^ Adjusted for age at diagnosis (continuous), stage (early, late), histologic subtype (serous, endometrioid and clear cell, mucinous and other), grade (borderline, low, high), and platinum and/or taxane chemotherapy (yes, no or unknown)

^b^ Mutually adjusted models include both p52 and p65 (cytoplasmic or nuclear staining)

^c^ Model does not include adjustment for grade or histologic subtype because only high-grade serous cases (*N* = 118) were included in this analysis

To evaluate if effects of the canonical and non-canonical NF-κB pathways were independent, we first combined cytoplasmic or nuclear staining for each factor and then included both p52 and p65 in mutually adjusted regression models (Table 3). Similar to analyses of separate cytoplasmic and nuclear staining, adjustment for age, stage, histologic subtype, grade, and chemotherapy treatment did not attenuate significance of associations for cytoplasmic or nuclear p52 with PFS (HR 1.53, 95% CI 1.08–2.19, *p* = 0.018) or OS (HR 1.61, 95% CI 1.12–2.33, *p* = 0.011). In contrast, higher cytoplasmic or nuclear p65 was not significantly associated with PFS or OS after the same statistical adjustment. When both p52 and p65 were included in mutually adjusted regression models, survival associations with p52 retained significance (PFS: HR 1.70, 95% CI 1.21–2.38, *p* = 0.002; OS: HR 1.64, 95% CI 1.16–2.32, *p* = 0.005) while associations with p65 remained non-significant.

Given that ovarian cancer is a highly heterogenous disease with five main subtypes with distinct prognoses, we additionally conducted analyses among the most clinically common subtype, high-grade serous cases (Table 3) [7]. In univariate analyses, high cytoplasmic or nuclear p52 was associated with significantly higher risks of disease progression (HR 1.79, 95% CI 1.17–2.75, *p* = 0.007) and death (HR 1.65, 95% CI 1.07–2.56, *p* = 0.024), while high p65 expression was not significantly associated with survival outcomes. Adjustment for age, stage, and chemotherapy treatment did not attenuate significance of associations for cytoplasmic or nuclear p52 with PFS (HR 1.68, 95% CI 1.08–2.62, *p* = 0.020) or OS (HR 1.61, 95% CI 1.03–2.53, *p* = 0.038), while associations with p65 remained non-significant. When both p52 and p65 were included in mutually adjusted regression models among high-grade serous cases, survival associations with p52 retained significance (PFS: HR 1.91, 95% CI 1.23–2.96, *p* = 0.004; OS: HR 1.70, 95% CI 1.09–2.66, *p* = 0.021). Further, in mutually adjusted regression models including adjustment for age, stage, and treatment, these associations with p52 and worse survival remained significant.

### Sensitivity analysis

Moreover, because ovarian cancer is a diverse clinical entity, we examined the robustness of our findings from analyses conducted after excluding specific subsets of cases (Table 4). Regardless of which cases were retained, results were materially unaltered with significant associations with PFS and OS for p52, including among chemotherapy naïve cases (*N* = 190, PFS and OS *p* < 0.001), invasive cases (*N* = 177, PFS *p* = 0.002, OS *p* = 0.007), serous cases (*N* = 143, PFS *p* < 0.001, OS p = 0.002), late stage cases (*N* = 137, PFS *p* = 0.009, OS *p* = 0.014), invasive serous cases (*N* = 131, PFS and OS *p* < 0.005), late stage serous cases (*N* = 119, PFS *p* = 0.005, OS *p* = 0.008) and high-grade serous cases (*N* = 118, PFS *p* = 0.005, OS *p* = 0.018). No associations reached statistical significance in sensitivity analyses for p65.

**Table 4:** Sensitivity analysis: NF-κB staining and ovarian tumor survival outcomes, the VUMC TROC

	Progression-free survival (PFS)		Overall survival (OS)	
	HR (95% CI) ^**a**^	***P***-value	HR (95% CI) ^**a**^	***P***-value
**Nuclear or cytoplasmic p52**				
Among all cases (*N* = 196)	**1.94 (1.39–2.71)**	**< 0.001**	**1.73 (1.24–2.43)**	**0.001**
Among chemotherapy naïve cases (*N* = 190)	**2.00 (1.42–2.81)**	**< 0.001**	**1.83 (1.30–2.58)**	**< 0.001**
Among invasive cases (*N* = 177)	**1.71 (1.21–2.42)**	**0.002**	**1.61 (1.14–2.29)**	**0.007**
Among serous cases (*N* = 143)	**1.98 (1.34–2.92)**	**< 0.001**	**1.90 (1.27–2.83)**	**0.002**
Among late stage cases (*N* = 137)	**1.68 (1.14–2.47)**	**0.009**	**1.63 (1.10–2.41)**	**0.014**
Among invasive serous cases (*N* = 131)	**1.89 (1.27–2.83)**	**0.002**	**1.91 (1.26–2.89)**	**0.003**
Among late stage serous cases (*N* = 119)	**1.84 (1.21–2.81)**	**0.005**	**1.81 (1.17–2.80)**	**0.008**
Among high-grade serous cases (*N* = 118)	**1.87 (1.21–2.87)**	**0.005**	**1.69 (1.09–2.62)**	**0.018**
**Nuclear or cytoplasmic p65**				
Among all cases (*N* = 196)	1.31 (0.94–1.82)	0.113	1.30 (0.93–1.81)	0.127
Among chemotherapy naïve cases (*N* = 190)	1.31 (0.93–1.83)	0.118	1.31 (0.93–1.85)	0.117
Among invasive cases (*N* = 177)	1.33 (0.95–1.88)	0.100	1.33 (0.94–1.89)	0.108
Among serous cases (*N* = 143)	1.04 (0.71–1.52)	0.860	1.20 (0.82–1.78)	0.352
Among late stage cases (*N* = 137)	1.34 (0.93–1.93)	0.119	1.23 (0.85–1.77)	0.265
Among invasive serous cases (*N* = 131)	1.15 (0.78–1.69)	0.482	1.30 (0.88–1.94)	0.193
Among late stage serous cases (*N* = 119)	1.33 (0.89–1.99)	0.160	1.29 (0.86–1.93)	0.214
Among high-grade serous cases (*N* = 118)	0.96 (0.63–1.47)	0.841	1.09 (0.71–1.69)	0.168

^a^ Adjusted for age at diagnosis (continuous)

## Discussion

This retrospective cohort study was conducted to test our hypothesis that both canonical and non-canonical NF-κB transcription factors influence epithelial ovarian cancer survival outcomes. We measured cytoplasmic and nuclear expression of p52 and p65 in tissue samples from 196 primary ovarian tumors from the Vanderbilt TROC, and tested associations with patient outcomes. We found that high cytoplasmic and nuclear expression of p52 was associated with worse PFS and OS in both crude and multivariable adjusted models. Nuclear p65 was only associated with worse PFS in unadjusted models. High cytoplasmic and nuclear p52 was independently associated with higher risk of disease progression and death, even in regression models that included adjustment for clinical covariates and p65 expression. Further, these associations remained significant when analyses were limited to high-grade serous ovarian cancer cases.

In line with our findings, prior studies have shown constitutive activation and overexpression of p65, a major marker of canonical NF-κB signaling, in many human epithelial cancers including ovarian, and have also demonstrated increasing expression of p65 along the continuum of borderline ovarian tumors to invasive carcinomas [43, 48–54]. However, evaluations of the relationship between p65 expression and ovarian cancer prognosis have had inconsistent findings [20–25]. Three small studies with 33, 68, and 85 epithelial cases found that those with high cytoplasmic, nuclear, or total p65 expression, respectively, tended to have worse PFS and/or OS [20, 22, 23]. In the largest of these three studies, the unadjusted OS association was significant, but was attenuated in multivariable adjusted models [23]. In a larger study of 114 serous ovarian cancer cases, those with high nuclear p65 expression had significantly worse PFS [24]. However, in another study among 324 high-grade serous ovarian cancer cases, high nuclear expression was associated with significantly better OS [21]. Reasons for these discordant findings likely include differences as to whether multivariable adjustment was conducted, what clinical covariates were adjusted for, and how p65 expression was categorized. For example, the threshold for dichotomizing expression as positive or high ranged from 21% of cells with moderate staining to 76% of cells with any staining, and was even selected to optimize survival differences in one study [20–22, 24]. In the current study, we dichotomized expression based on median values, and found that higher cytoplasmic p65 was not significantly associated with PFS or OS, while higher nuclear p65 was only significantly associated with worse PFS in crude models. The association between higher nuclear p65 and worse OS missed significance in crude models (*p* = 0.052). Following multivariable adjustment, neither cytoplasmic nor nuclear p65 were associated with survival outcomes. Critically, in models with mutual adjustment for both p52 and p65 expression, associations with OS and PFS were attenuated for p65, indicating that prior associations with the canonical pathway may be mediated by p52 and the non-canonical NF-κB pathway.

To our knowledge, this is the first study to assess the relationship between expression of p52, a major component of the non-canonical NF-κB pathway, and epithelial ovarian cancer prognosis, particularly high-grade serous ovarian cancer. We found that higher p52 was associated with higher risk of disease progression and death, and that these associations were robust to multivariable adjustment, including for expression of p65. While canonical and non-canonical NF-κB signaling are known to have some cross-talk, our mutually adjusted analysis indicates that the effects of p52, a primary contributor to the non-canonical pathway, on ovarian cancer outcomes are independent of canonical NF-κB signaling through p65 expression. We also found that correlations between p52 and p65 expression levels were low, suggesting that these two pathways are not highly inter-connected in epithelial ovarian tumors. While there are common factors known to regulate both pathways in cancer cells to support tumorigenesis, such as IKKα, in general there is limited information about how the two pathways may overlap in ovarian cancer or in other cancer cell types. By contrast, there is stronger evidence showing that each pathway can individually influence cancer cell behavior and promote tumorigenesis across the spectrum from borderline tumors to invasive carcinomas, dependent on factors such as tumor type, localization, and microenvironment [43, 55, 56].

While this is the first study to demonstrate an association with clinical disease prognosis, several additional lines of mechanistic evidence support a pro-tumorigenic role for non-canonical NF-κB signaling independent of the canonical pathway [35]. We previously found that downregulation of cyclooxygenase-1 (COX-1) expression, an established pro-tumor mediator in ovarian cancer, resulted in reduced expression of multiple non-canonical NF-κB signaling components, including *RELB*, *NFKB2* (p100/p52), *CHUK* (IKKα), *and MAP3K14* (NF-κB-inducing kinase); in contrast, expression of canonical NF-κB pathway members was not changed by COX-1 knockdown [57]. Moreover, overexpression of p52 promotes lung tumorigenesis in mice, and associations have been demonstrated between non-canonical NF-κB signaling, p52-gene targets, and worse prognosis in human lung adenocarcinoma cases [33]. Further studies of in vitro and in vivo models of ovarian cancer have shown that non-canonical NF-κB signaling promotes cell growth and tumorigenicity as well as cancer stem cell self-renewal [31, 32, 34, 35]. Aberrant expression of tissue transglutaminase in ovarian tumors has been implicated as an upstream regulator of p52 expression and non-canonical signaling, ultimately contributing to disease progression and intraperitoneal metastasis [31]. Over-expression of NIK has also been demonstrated in human ovarian cancer cell lines, leading to downstream activation of non-canonical signaling and increased tumorigenicity [32]. Known gene targets of non-canonical NF-κB signaling include important mediators of cell proliferation, such as cyclin D1; cell survival, such as Bcl-x; and tumor invasion and vascular growth, such as MMP-9 and VEGF, all of which are frequently deregulated in cancer cells, contribute to epithelial-to-mesenchymal transition, and have been associated with poor prognosis [58–60].

Additionally, NF-κB pathway activation is known to contribute to inflammation in the tumor microenvironment [18, 19, 61]. Studies in the breast cancer context have indeed demonstrated associations between expression of NF-κB and increased stromal tumor contribution [54]. Although this study focused on epithelial expression of NF-κB transcription factors, future studies should utilize tissue microarrays enriched for stromal compartments to analyze associations of NF-κB signaling with immune infiltration, as well as NF-κB signaling within immune or stromal cells. Given increasing interest in immunotherapy for treatment of cancer, these data may provide insights into mechanisms of disease progression or potential treatment strategies combining immunotherapy with NF-κB modulators. While the canonical NF-κB pathway has been targeted in several solid tumor types using various non-specific NF-κB inhibitors such as bortezomib, thymoquinone, and curcumin, the clinical potential of targeting non-canonical signaling in cancer remains unknown [62–64].

Strengths of this study include assessment of both nuclear and cytoplasmic p52 and p65 staining in tumors, multivariable adjusted regression models, and mutual adjustment to parse out separate effects of canonical and non-canonical NF-κB pathway member expression. We additionally performed sensitivity analysis to assess the robustness of our findings, which were materially unaltered among chemotherapy naïve, invasive, serous, late stage, invasive serous, late stage serous, and high-grade serous cases. Our primary limitation was sample size; with only 196 tumor samples stained, we were unable to evaluate associations among all five major subtypes, which are known to have distinct etiologies and prognoses; we were able to conduct analyses among the most clinically common subtype, high-grade serous ovarian cancer, which demonstrated significant associations between p52 expression and disease prognosis [7]. Another possible limitation is that nuclear expression was markedly lower than cytoplasmic expression. However, we dichotomized based on median values, and created combined indices for high nuclear or cytoplasmic staining as in prior studies of NF-κB elements to maximize the generalizability of our results [23]. Further, while nuclear p52 is generally considered to represent non-canonical NF-κB pathway activity, p52 may form heterodimers with all other NF-κB pathway members [65]. While immunohistochemical detection of RelB could also provide information on non-canonical NF-κB pathway activity, a prior study of RelB in ovarian tissue only demonstrated cytoplasmic staining [20]. Further, RelB is highly unstable and is known to preferentially complex with p52 [66]. Therefore, this study focused on evaluation of associations between epithelial ovarian cancer survival and expression of p52, a major component of the non-canonical pathway about which there is limited information. Finally, we recognize that our study population was predominantly White and from a single center, potentially further limiting the generalizability of our study.

## Conclusions

In conclusion, we have shown that expression of p52, a major mediator of non-canonical NF-κB signaling, is an important prognostic factor for high-grade serous ovarian cancer, and that this association is independent of canonical NF-κB signaling through p65. While prior work has demonstrated potential mechanisms by which non-canonical NF-κB signaling may contribute to ovarian cancer pathogenesis, this data provides a crucial clinical link that expression of a non-canonical NF-κB transcription factor is associated with ovarian cancer prognosis, and interventions to inhibit non-canonical NF-κB signaling should be explored as novel therapies to limit ovarian cancer progression and optimize survival outcomes.

## Supplementary information

**Additional file 1.** Flow Chart of Patient Inclusion/Exclusion. Description of data: Flow chart demonstrating inclusion and exclusion of primary ovarian tumor tissue samples from patients undergoing staging and/or cytoreductive surgery at VUMC between 1994 and 2004.

## Data Availability

All datasets generated during and analyzed during this study will be made available after garnering institutional approval and enacting appropriate data sharing agreements.

## Abbreviations

CI: Confidence Intervals
COX-1: Cyclooxygenase-1
EMR: Electronic Medical Record
FIGO: International Federation of Gynecology and Obstetrics
HR: Hazard Ratio
IHC: Immunohistochemistry
IkB: Inhibitor of KappaB
IKK: Inhibitor of KappaB Kinase
MMP-9: Matrix Metalloproteinase-9
NF-kB: Nuclear Factor-KappaB
NIK: Nuclear Factor-KappaB-Inducing Kinase
OS: Overall Survival
PARPi: Poly ADP Ribose Polymerase Inhibitors
PFS: Progression-Free Survival
RECIST: Response Evaluation Criteria in Solid Tumors
TMA: Tissue Microarray
TROC: Tissue Repository for Ovarian Cancer
VEGF: Vascular Endothelial Growth Factor
VUMC: Vanderbilt University Medical Center
WHO: World Health Organization
