# Supplementary material for: Expression of p52, a non-canonical NF-kappaB transcription factor, is associated with poor ovarian cancer prognosis
Source: Biomark Res. 2020 Sep 15;8:45. doi: 10.1186/s40364-020-00227-y (PMC7493985; doi:10.1186/s40364-020-00227-y)
Supplement: Supplementary file 1 — Additional file 1. Flow Chart of Patient Inclusion/Exclusion. Description of data: Flow chart demonstrating inclusion and exclusion of primary ovarian tumor tissue samples from patients undergoing staging and/or cytoreductive surgery at VUMC between 1994 and 2004. [file 40364_2020_227_MOESM1_ESM.pptx]

## Slide 1
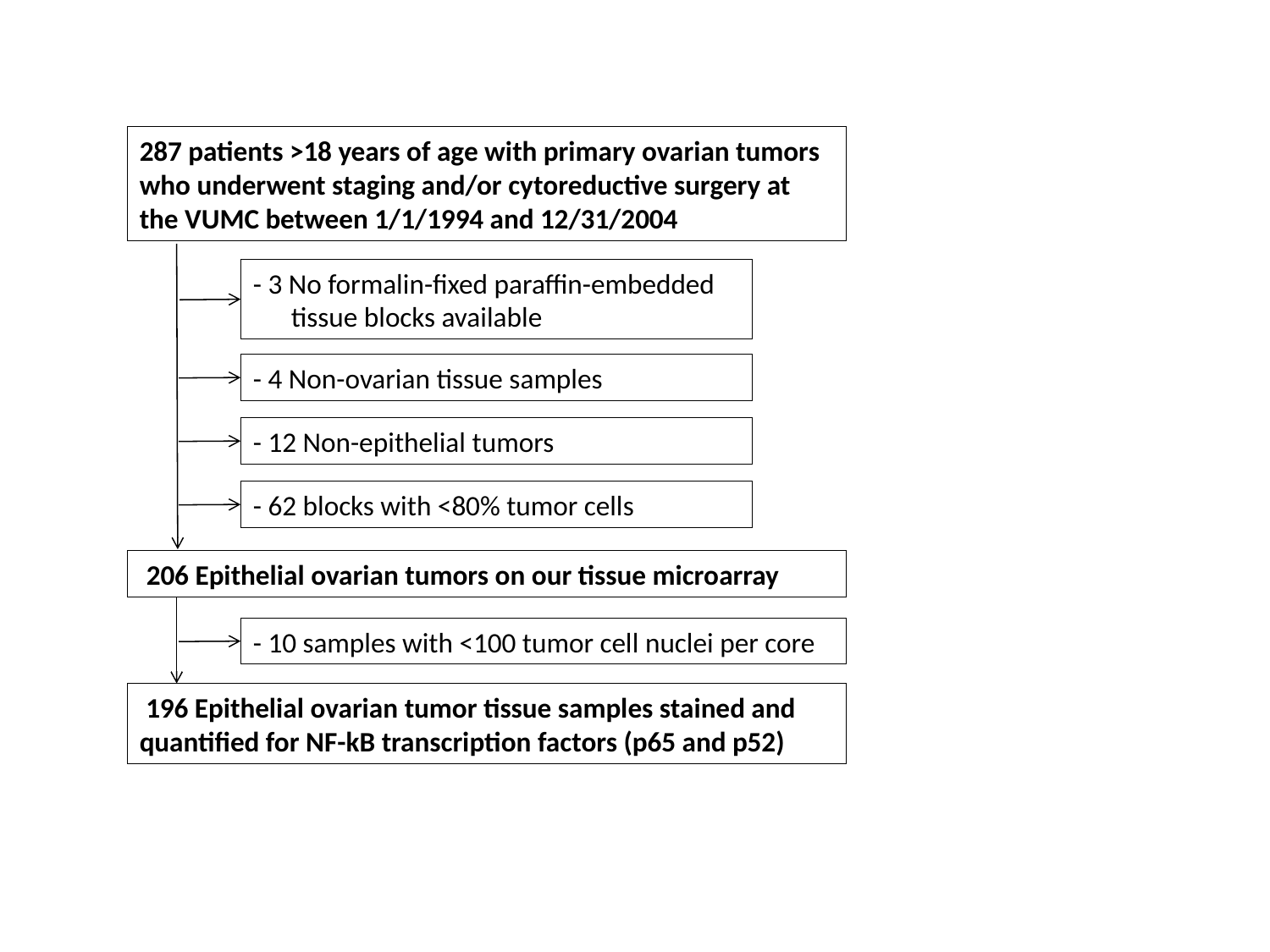

287 patients >18 years of age with primary ovarian tumors who underwent staging and/or cytoreductive surgery at the VUMC between 1/1/1994 and 12/31/2004​
- 3 No formalin-fixed paraffin-embedded
 tissue blocks available
- 4 Non-ovarian tissue samples
- 12 Non-epithelial tumors
- 62 blocks with <80% tumor cells
 206 Epithelial ovarian tumors on our tissue microarray
- 10 samples with <100 tumor cell nuclei per core
 196 Epithelial ovarian tumor tissue samples stained and quantified for NF-kB transcription factors (p65 and p52)
